# Regulation of nitrogen transformation and microbial community by inoculation during livestock manure composting

**DOI:** 10.1111/1758-2229.13256

**Published:** 2024-04-04

**Authors:** Rui Cao, Yihao Huang, Ruyu Li, Ke Li, Zhuqing Ren, Jian Wu

**Affiliations:** ^1^ Key Laboratory of Agriculture Animal Genetics, Breeding and Reproduction of the Ministry of Education & Key Laboratory of Swine Genetics and Breeding of the Ministry of Agriculture and Rural Affairs, College of Animal Science Huazhong Agricultural University Wuhan China; ^2^ Frontiers Science Center for Animal Breeding and Sustainable Production Wuhan China; ^3^ Hubei Hongshan Laboratory Wuhan China

## Abstract

This study examined the effects of three *Bacillus* strains and one *Saccharomyces cerevisiae* strain on nitrogen transformation and microbial communities in pig and chicken manure compost. The findings revealed that the use of compound microbial inoculants increased the compost temperature, accelerated moisture reduction, enhanced cellulase activity, and stimulated the accumulation of NH_4_^+^‐N, NO_3_^−^‐N, and total nitrogen (TN), resulting in a 9% increase in TN content. The abundance of Firmicutes decreased by 3.95% at the maturation phase, while Actinobacteria and Bacteroidetes increased by 1.64% and 1.85%, respectively. Inoculation led to an increase in *amoA*, *nxrA* and *nifH* gene copy numbers, while simultaneously reducing the abundance of *nirK*, *nosZ* and *nirS* genes. It also resulted in an increase in functional enzyme levels, specifically nif and amo, with a corresponding decrease in nor. *Clostridium*, *Phascolarctobacterium*, *Eubacterium* and *Faecalibacterium* from the class Clostridium, which have a significant correlation with *nifH* and *nxrA* genes, suggest their likely crucial role in nitrogen retention and fixation. Inoculation aided in the removal of pathogenic bacteria and antibiotic resistance genes (ARGs) like fluoroquinolones, nucleosides and nitroimidazole. This study provides effective theoretical support for the mechanism of nitrogen retention and fixation, and for improving the quality of compost.

## Graphical abstract

This study investigates the application of specific microbial inoculants in the composting process of pig and chicken manure, resulting in a 9% increase in total nitrogen content. The introduction of these inoculants instigated a beneficial shift in the microbial community, augmenting nitrogen retention and fixation, while concurrently diminishing the prevalence of pathogens and antibiotic resistance genes. This research highlights the potential of microbial interventions to enhance the quality of agricultural compost and mitigate environmental risks.

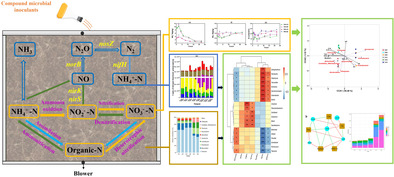

## INTRODUCTION

Due to the rapid advancement of intensive farming practices, livestock manure has emerged as the primary contributor to non‐point source pollution in agriculture. The large emissions, high difficulty in treatment, and low utilization rate of livestock manure have seriously affected the health and production of livestock, become a bottleneck restricting the sustainable development of animal husbandry, and also brought great pressure to the ecological environment governance. Livestock manure is rich in essential nutrients, including nitrogen, phosphorus, and potassium (Bai et al., 2016), which may lead to substantial nutrient losses if not fully harnessed. Aerobic composting, as highlighted by (Mengqi, Shi, Ajmal, Ye, & Awais, 2021) and (Ravindran et al., 2019), stands as an effective method for achieving the reduction, harmless treatment, and resource utilization of livestock manure. Nevertheless, the ongoing metabolic activities of microorganisms during aerobic composting result in substantial nitrogen loss through gas emissions. This significant nitrogen loss substantially impacts both the composting process and the quality of the end compost product, ultimately leading to severe air pollution. The process of nitrogen cycling and transformation within the compost directly influences the extent of nitrogen loss. The processes of microbial nitrification and denitrification are widely recognized as the primary driving forces governing the nitrogen cycle (Cáceres, Malińska, & Marfà, 2018). Ammonia‐oxidizing bacteria secrete ammonia oxidase (AMO) to initiate nitrification in the nitrogen cycle, which converts ammonia to stable nitrates (Yamamoto, Oishi, Suyama, Tada, & Nakai, 2012). Studies have shown that the *nxrA* gene is the main functional gene that reflects the intensity of nitrification (Yin et al., 2020), some studies have used *nxrA* as a key gene for detecting nitrite‐oxidizing bacteria in compost (Li, Guo, Lu, Shan, & Huang, 2016; Wu et al., 2018). Inhibition of N_2_O emission could reduce nitrogen loss. A positive correlation has been found between N_2_O emissions and the *nosZ* gene in denitrifying bacteria (Chen et al., 2017; Guo et al., 2020). In addition, nitrogen fixation can reduce nitrogen to organic nitrogen, and the nitrogenase gene *nifH* is considered to be a specific marker gene for nitrogen‐fixing microorganisms (Kuypers, Marchant, & Kartal, 2018). Nitrogen transformation is ultimately completed by ammonifying bacteria, nitrifying bacteria, and denitrifying bacteria. Hence, it is imperative to investigate the pivotal functional genes and dynamics of microbial communities involved in nitrogen transformation during composting.

Adding adsorbents and microbial inoculums to compost have a positive effect on reducing nitrogen loss during composting (Mao et al., 2018; Wang et al., 2017). The incorporation of zeolite and biochar during pig manure composting has been demonstrated to significantly mitigate NH_3_ (by 63.40%) and N_2_O (by 78.13%) emissions, as evidenced by scientific studies (Wang et al., 2017). But the adsorbent is relatively expensive and some adsorbent materials may cause secondary pollution. Numerous studies have demonstrated that exogenous microbial agents play a pivotal role in facilitating the succession of microbial communities, enhancing the composting process, and augmenting both the efficiency and quality of compost (Liu et al., 2021; Wang et al., 2021; Yang et al., 2018). Mao et al. (2018) found that adding microbial powder enhanced nitrogen storage and microbial community using a laboratory‐scale reactor for composting. Guo et al. (2020) found that the addition of *Bacillus megatherium* can promote the ammonia oxidation process in compost and reduce the ammonia emission. Studies have found that the addition of *Lactobacillus plantarum* can improve the efficiency and quality of sheep manure compost (Li et al., 2020).

Previous studies predominantly focused on assessing the efficacy of a singular functional strain in composting (Duan et al., 2019; Duan et al., 2020; Yong et al., 2011). There was a lack of systematic and in‐depth research on the mechanism. Our previous study revealed *Bacillus* as the predominant bacteria during the high‐temperature phase of primary composting (Yi et al., 2012), the compound microbial inoculum accelerated the process of compost and had a nitrogen fixation effect during the pig manure composting (Li et al., 2020). However, it is not clear how exogenous microorganisms promote nitrogen cycling to control nitrogen loss. In this study, the effects of compound microbial inoculums on functional genes of nitrogen cycling, material transformation, and microbial community in compost were investigated, which will provide a theoretical basis for the mechanism of nitrogen conservation and fixation and the improvement of compost efficiency and quality.

## EXPERIMENTAL PROCEDURES

### Raw compost materials

The raw materials for the composting experiment were pig manure, chicken manure, and sawdust. The pig manure used in the compost came from the fine pig farm of Huazhong Agricultural University, the chicken manure was from Wuhan Chaotuo Ecological Agriculture Co., Ltd., and the sawdust was from the Veterinary Hospital of Huazhong Agricultural University. The contents of total carbon, total nitrogen, and water in the initial swine manure were 37.94%, 2.63% and 69.25%, respectively. The contents of total carbon, total nitrogen, and water in chicken manure were 30.27%, 3.47%, and 71.69%, respectively. In sawdust, there was 45.22% carbon, 0.36% nitrogen, and 10.45% water. Among them, the content of total carbon (TC) and TN is based on dry weight.

### Composting device and process

This experiment simulated the composting mode of the reactor. All the experiments were carried out in a 60 L rectangular foam container. The composting device was consistent with our previous studies (Li, Cao, et al., 2020). The bottom of the foam container was covered with about 3 cm of sawdust as a buffer layer, and one ventilation hole was opened at the side to facilitate temperature measurement. The ventilation device was used for regular ventilation to ensure a uniform ventilation volume.

The compost experiments were divided into four groups and were labelled as CM + CK (chicken manure + sawdust), CM + AB (chicken manure + sawdust + 1% compound microbial inoculums), PM + CK (swine manure + sawdust), and PM + AB (swine manure + sawdust + 1% compound microbial solution) (Li, Cao, et al., 2020). The material ratio at the initial stage of composting was chicken manure: sawdust = 5:1; When the ratio of swine manure to sawdust was 7:1. The C/N ratios of the four groups were all adjusted to about 20, and the water content was about 65%. Before composting, each experimental group was inoculated with 1% compound microbial solution (*Bacillus subtilis*: *Bacillus amyloliquefaciens*: *Bacillus licheniformis*: *Saccharomyces cerevisiae* = 3:1:3:4). The aforementioned strains were derived from previous samples of swine manure compost. The strain *Bacillus amyloliquefaciens* has been saved in the China Center for Type Culture Collection under the No. M2020098. Samples are collected according to the temperature variation of the compost, and stored in refrigerators at 4°C and −20°C for later use.

### Detection of the physicochemical index

The temperature of compost and the ambient temperature were measured at 9:00 AM and 3:00 PM daily using a Kaitai B‐8A thermometer. Moisture content was measured according to the difference in sample dry weight. Cellulase activity (S‐CL) of the samples was determined by a soil cellulase extraction kit (Sorebo, Beijing). The TN and TC of the samples were analysed by a Varimax‐CN Germany Elemental. The pH was measured by a PHS‐3C pH meter. The NH_4_^+^‐N and NO_3_^−^‐N were analysed by an ammonia, nitrogen and total phosphorus determinator (LH3‐CNP) and an ultraviolet spectrophotometer (722E), respectively.

### Microbial DNA extraction and qPCR

In accordance with previous research, we extracted microbial DNA and performed qPCR under the same conditions (Li, Liu, et al., 2020). The qPCR primers were shown in Table 1. The standard curve of qPCR was generated using the plasmid carrying the target gene with the correlation coefficient *R* ^2^ >0.99, and the copy number of the nitrogen transformed gene was calculated by external reference method from the standard curve.

**TABLE 1:** PCR primer sequences and annealing temperature.

Gene	Forward (5′–3′)	Reverse (5′–3′)	AT (°C)
*16S rDNA*	CCTACGGGAGGCAGCAG	ATTACCGCGGCTGCTGG	55
*nifH*	TGCGAYCCSAARGCBGACTC	ATSGCCATCATYTCRCCGGA	58
*nxrA*	CAGACCGACGTGTGCGAAAG	TCYACAAGGAACGGAAGGTC	56
*amoA*	GGGGTTTCTACTGGTGGT	CCCCTCKGSAAAGCCTTCTTC	56
*narG*	TA(CT)GT(GC)GGGCAGGA(AG)AAACTG	CGTAGAAGAAGCTGGTGCTGTT	58
*nirK*	ATCATGGTSCTGCCGCG	GCCTCGATCAGRTTGTGGTT	57
*nirS*	GT(C/G)AACGT(C/G)AAGGA(A/G)AC(C/G)GG	GA(C/G)TTCGG(A/G)TG(C/G)GTCTTGA	57
*nosZ*	CCCGCTGCACACCRCCTT CGA	CGTCGCCSGAGATGTCGA TCA	56

### Metagenomic sequencing

The six samples of swine manure compost with compound microbial inoculum (AB) and blank control (CK) were collected, named CK1, AB1, CK2, AB2, CK3 and AB3, were used to analyse changes in microbial community structure, functional characteristics, and evolutionary relationships by performing metagenomic sequencing. In the swine manure compost process, day 1 reflects the mesophilic phase, day 2 reflects the thermophilic phase, and day 22 reflects the maturation phase. A total of 18 samples were included in the study, with three replicates for each group. In accordance with Illumina's standard protocol, samples were tested for quality, libraries were constructed, libraries were tested for quality, and library sequences were performed. The specific process was as follows: genomic DNA of the compost sample was extracted and tested qualified. The DNA was mechanically disrupted (using ultrasonic interruption) to induce fragmentation, followed by purification of the resulting fragments. Subsequently, repair was performed at the ends, addition of an A residue at the 3′ end, and connection with a sequencing adapter. Fragment size selection was achieved through agarose gel electrophoresis, leading to the formation of a sequencing library via PCR amplification. Qualitative control of the sequencing reads was performed by Illumina. Clean reads were filtered for subsequent bioinformatics analysis. Clean reads were spliced and assembled to predict coding genes, and functional annotation of coding genes were performed in general and special databases. At the same time, taxonomic analysis of Clean reads was conducted to collect the species composition and abundance information of the sample, and the conclusion report was completed by BMK cloud.

### Statistical analysis

Microsoft Excel 2010 was used to determine the mean and standard deviation of physical and chemical indexes and nitrogen transformation genes. GraphPad Prism 7 was used for image rendering. SPSS 25.0 statistical software package was used for statistical and significance difference analysis. Network analysis was performed using Cytoscape and Adobe Illustrator CS5. The tool software RGI (Version 4.2.2) in CARD database was used to compare the protein sequences of non‐redundant genes with the database, and the corresponding resistance gene related information was obtained. Diamond software (Version 0.9.24) was used to compare the protein sequences of non‐redundant gene sets with the KEGG database to obtain the pathway map. Diamond software (Version 0.9.24) was used to compare the protein sequences of the non‐redundant gene set with the Nr database to obtain the species composition and relative abundance information of the samples for statistical analysis.

## RESULTS

### Changes of composting indicators

**FIGURE 1:**
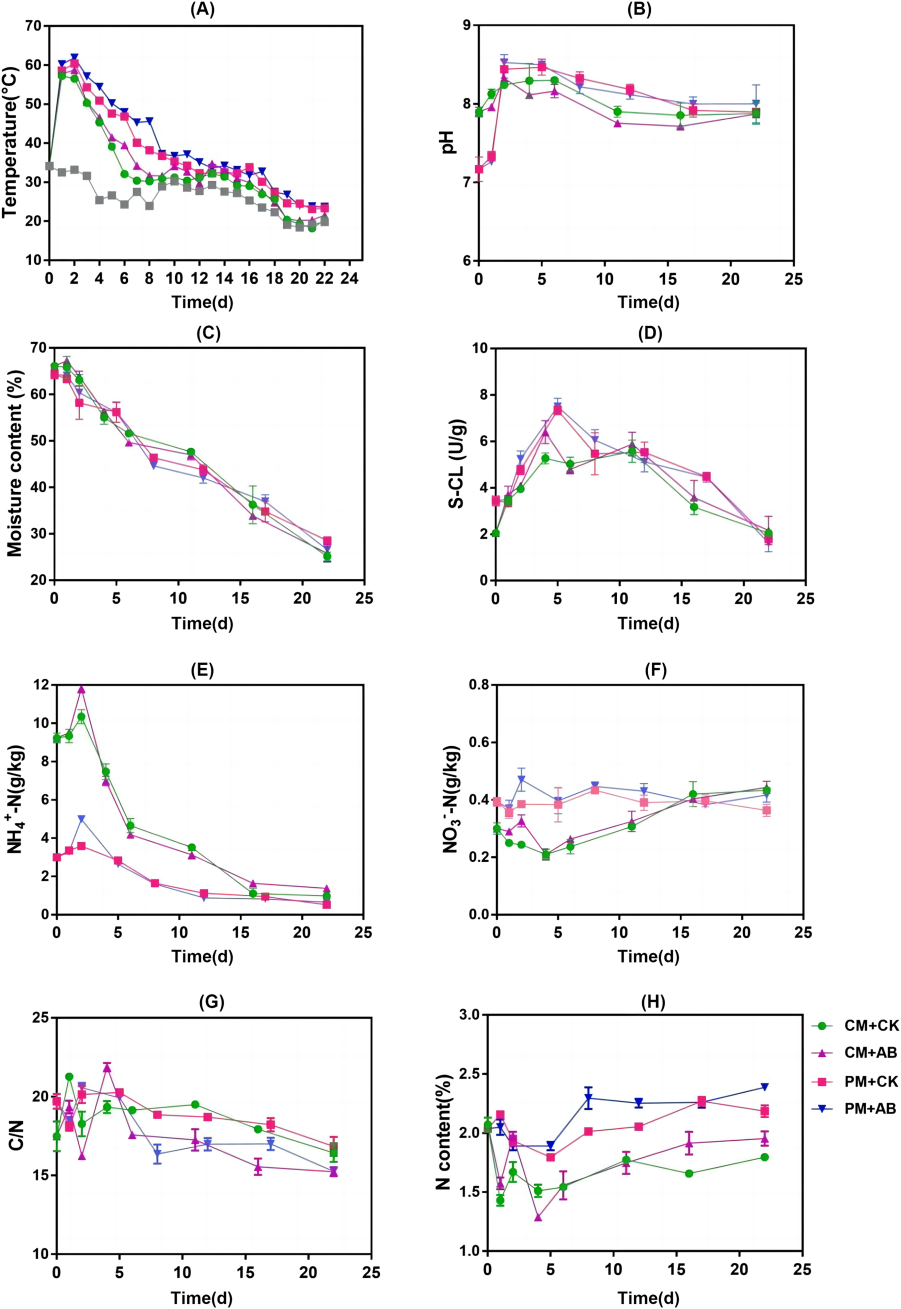
Physicochemical changes during livestock manure composting (A) Temperature; (B) Moisture; (C) pH; (D) Cellulase activity; (E) NH_4_^+^‐N; (F) NO_3_^−^‐N; (G) C/N; (H) TN. PM + CK: The swine manure compost; PM + AB: Add 1% compound microbial inoculum during swine manure composting; CM + CK: The chicken manure compost; CM + AB: Add 1% compound microbial inoculum during chicken manure composting. The grey line indicates ambient temperature throughout the duration of the experiment.

The aerobic composting of swine manure and chicken manure lasted for a total duration of 22 days, during which the thermophilic phase persisted for more than 3 consecutive days, thereby satisfying the requirements stipulated by the reactor composting process (Figure 1A). The results showed that the composting reaction temperature could be increased by inoculating compound microbial. The highest temperature of the swine manure composting group (PM + AB) was 61.9°C, and the highest temperature of the chicken manure composting group (CM + AB) was 58.7°C. The ammoniation was stronger at the initial stage of composting, and the pH value gradually increased. The pH value gradually decreased and reached a stable range of 7–8 during the maturity stage (Figure 1B). Inoculation accelerated water loss (Figure 1C). The water content in PM + AB group decreased to 26.58% and in PM + CK group decreased to 28.56%. The cellulase level of PM + AB group reached the highest value of 7.5 U/g on the fifth day of composting, which was higher than 7.34 U/g in PM + CK group (Figure 1D), indicating that the cellulase activity in compost could be improved by microbial inoculation.

The NH_4_^+^‐N, NO_3_^−^‐N, and total nitrogen contents in the inoculum group were found to be higher than those in the control group at the completion of composting. NH_4_^+^‐N peaked on the second day of composting (the thermophilic phase), and the NH_4_^+^‐N content of pig manure inoculant group was 4.99 g/kg, which was much higher than that of pig manure control group (3.58 g/kg), and the NH_4_^+^‐N content of CM + AB group was 1.14 times that of chicken manure control group (Figure 1E), which indicated that inoculum could accelerate the degradation of organic substances and ammonification process. The NO_3_^−^‐N content of the inoculum group was significantly higher than that of the control group. The NO_3_^−^‐N content of CM + AB group was 1.5 times that of CM + CK group (Figure 1F), which indicated that inoculum promoted nitrification and NO_3_^−^‐N accumulation. The total nitrogen content in CM + CK group was 1.80 g/kg, and that in CM + AB group was 1.95 g/kg. The total nitrogen content in PM + CK group and PM + AB group was 2.18 and 2.39 g/kg, respectively indicating that inoculum played a certain role in nitrogen preservation and nitrogen fixation (Figure 1H). The C/N ratio of PM + AB group was 15.26, and that of PM + CK group was 16.85 (Figure 1G). The above results indicated that adding compound microbial inoculum could increase the temperature of the compost, accelerate the water loss and had the effect of nitrogen preservation and nitrogen fixation.

### Changes in abundance of functional genes for nitrogen transformation

**FIGURE 2:**
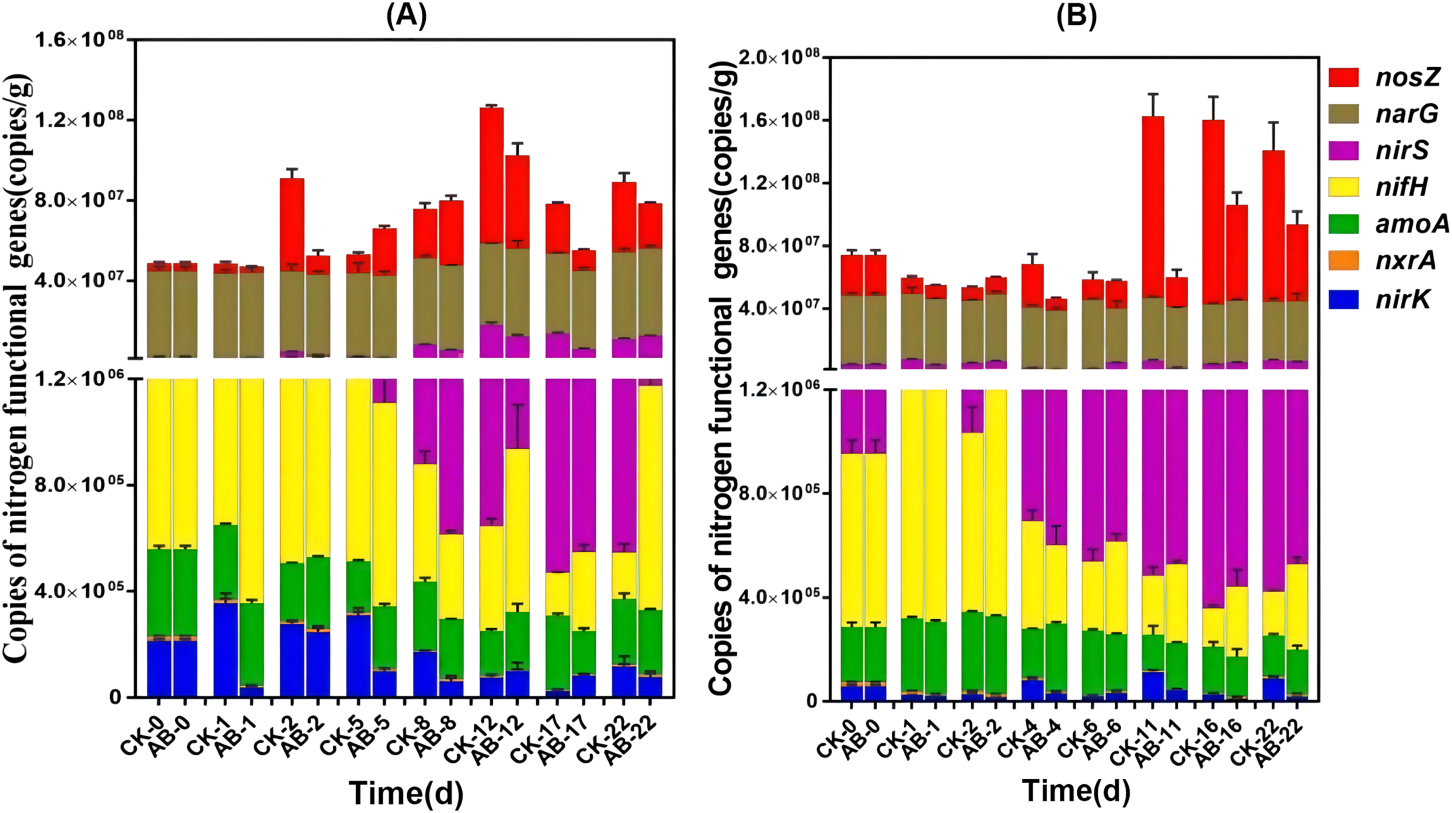
Abundance change of nitrogen transformation gene during swine and chicken manure composting. (A) The swine manure compost; (B) The chicken manure compost. CK: The control group of compost; AB: Add 1% compound microbial inoculum in compost. The numbers 0, 1, 4, 5, 6, 8, 11, 12, 16, 17 and 22 represent the days of compost, respectively.

The quantitative analysis of functional genes involved in nitrogen transformation, including *amoA* and *nxrA* for nitrification, *nirS*, *nirK*, *nosZ* and *narG* for denitrification, as well as the nitrogen‐fixing gene *nifH* in swine and chicken manure compost was performed using qPCR (Figure 2). Compost derived from swine manure showed a decline in amoA gene expression (Figure 2A), while the abundance of the *amoA* gene was higher in the group inoculated with compound microbial inoculum compared to the control group. These findings suggested that adding compound microbial inoculum facilitates NH_4_^+^‐N transformation and mitigates NH_3_ release. The *nxrA* gene is a marker gene of nitrification, which converts ammonia into stable nitrate to reduce nitrogen loss, but the proportion of *nxrA* gene was relatively low in this study. The abundance of *nxrA* gene in that initial stage of swine manure compost and chicken manure compost was 1.87 × 10^4^copy/g and 1.67 × 10^4^copy/g, respectively (Figure 2B). The change trend of *nxrA* gene was opposite in pig manure compost and chicken manure compost. Inoculation increased the abundance of *nxrA* gene in the mesophilic and maturation phase of pig manure and chicken manure compost, which was consistent with the change trend of nitrate nitrogen. The abundance of genes related to denitrification tended to increase as a whole, except for the abundance of *narG* gene, there were obvious differences in the expression of other genes. The abundance of *nirK*, *nirS*, *nosZ* genes in the swine manure compost and *nosZ* gene in the chicken manure compost showed an increasing trend, and compared with the control group, the inoculation group had significantly fewer genes.

The abundance of nitrogenase *nifH* gene was the highest, which remained in a high abundance state throughout the high temperature period of compost. Compared with the control group, the abundance of *nifH* gene was significantly increased by inoculation with microbial agent in both swine manure and chicken manure compost. In summary, the inoculation of microbial agent changed the abundance of nitrogen transformation functional genes, increased the abundance of *amoA*, *nxrA* and *nifH* genes, and decreased the expression abundance of *nirK*, *nosZ* and *nirS*. Adding compound microbial inoculum could promote nitrification and nitrogen fixation of microorganisms in compost, inhibit denitrification, reduce nitrogen loss, and play a role in nitrogen fixation and nitrogen conservation.

### Changes of microbial community in compost

**FIGURE 3:**
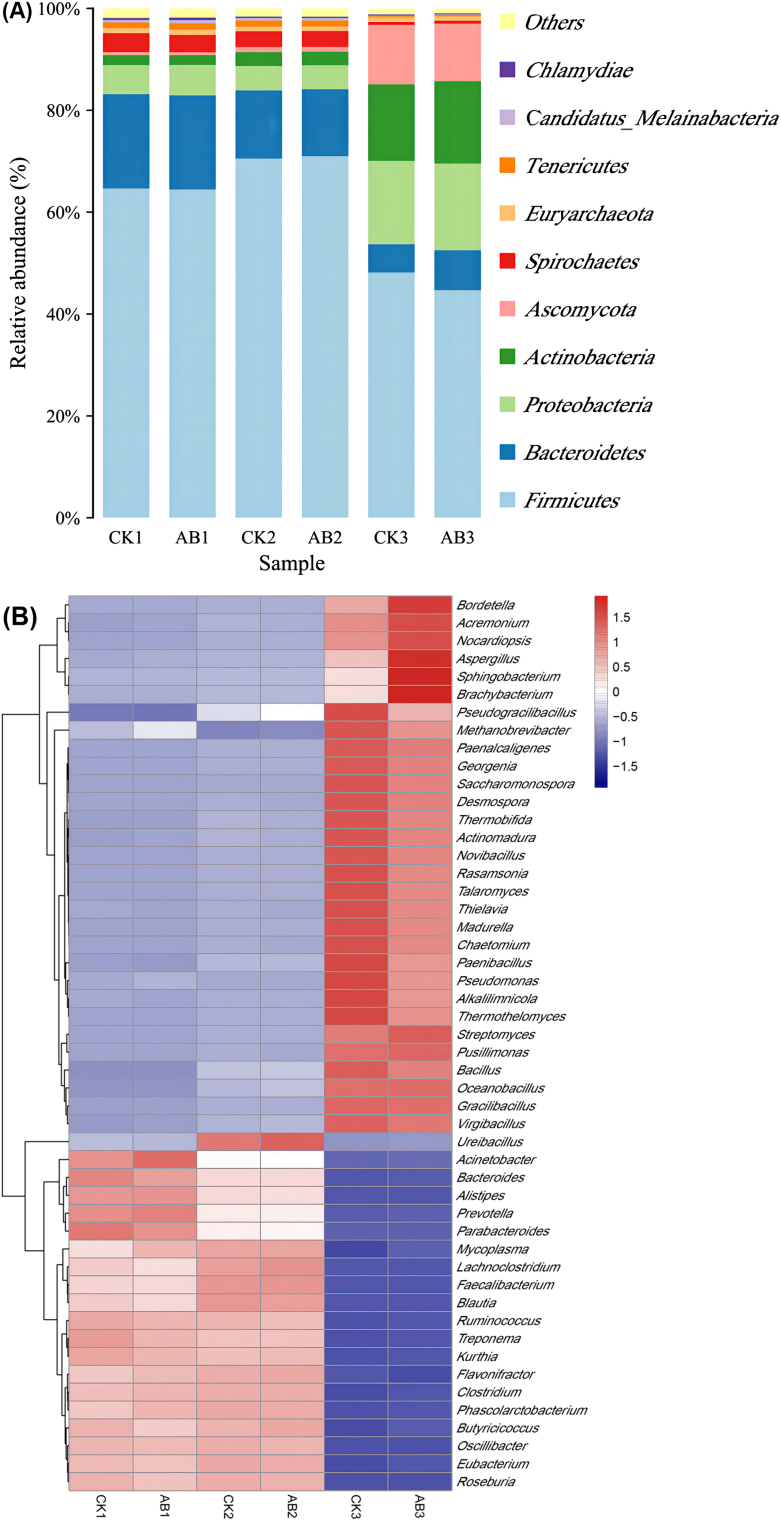
Structure and abundance of microbial community in swine manure compost. (A) Phylum level, (B) genus level; CK: The control group of swine manure composting; AB: Add 1% compound microbial inoculum in the swine manure composting. The numbers 1, 2 and 3 correspond to the mesophilic phase (day 1), thermophilic phase (day 2) and maturation phase (day 22) of swine manure composting, respectively.

The metagenomic sequencing raw data had been submitted to the NCBI SRA network shared database (http://www.ncbi.nlm.nih.gov/bioproject/684647; Submission ID: SUB8202970; BioProject ID: PRJNA684647). The dynamics of the microbial community at the phylum level during swine manure composting are illustrated in Figure 3A. *Firmicutes*, *Proteobacteria*, *Bacteroidetes*, *Actinobacteria*, *Spirochaetae* and *Ascomycota* were the dominant fungi. There was a strong association between *firmicutes* and mesophilic phase and thermophilic phase of swine manure composting, which was consistent with a number of research findings (Guo et al., 2020; Lei et al., 2021; Wang et al., 2016). Microbial inoculum decreased *Firmicute* abundance over the control group, as well. In the maturity stage of compost, *Firmicutes* gradually decreased, and the abundance of *Proteobacteria*, *Actinobacteria*, and *Ascomycota* increased, gradually replacing the dominant position of *Firmicute*, especially in the group with microbial inoculum. During the maturity stage of swine manure compost, the community structure exhibited a significant disparity between the group subjected to microbial inoculum and the control group. The abundance of *Firmicutes* was decreased by 3.95%, and the abundance of *Actinobacteria* and *Bacteroidetes* increased by 1.64% and 1.85%, respectively.

The changes in the microbial community at the genus level were concurrently examined, as depicted in Figure 3B. The microbial genera with higher abundance in the mesophilic phase were *Bacteroides*, *Acinetobacter*, *Prevotella* and *Parabacteroides*. Most of them were pathogenic bacteria. In the thermophilic phase, thermophilic bacteria such as *Ureibacillus*, *Blautia*, *Faecalibacterium* and *Lachnoclostridium* were dominant, and the abundance of thermophilic bacteria in the group with microbial inoculum was higher than that in the control group. In the maturation phase of compost, *Pseudomonas*, *Pseudogracilibacillus*, *Methanobrevibacter*, *Novibacillus* and *Alkalilimnicola* were dominant in the control group, which mainly belonged to *Firmicutes* and *Proteobacteria*. The relative abundance of *Sphingobacterium*, *Brachybacterium*, *Aspergillus*, *Acremonium* and *Bordetella* was the highest in the group with compound microbial inoculum, which mainly belonged to *Bacteroidetes*, *Actinobacteria* and *Fungi*. This was consistent with the change trend of phylum level. When compost reached its maturation phase, microbial communities of Bacteroidetes and Actinobacteria increased.

### Changes in functional enzymes of nitrogen transformation

**FIGURE 4:**
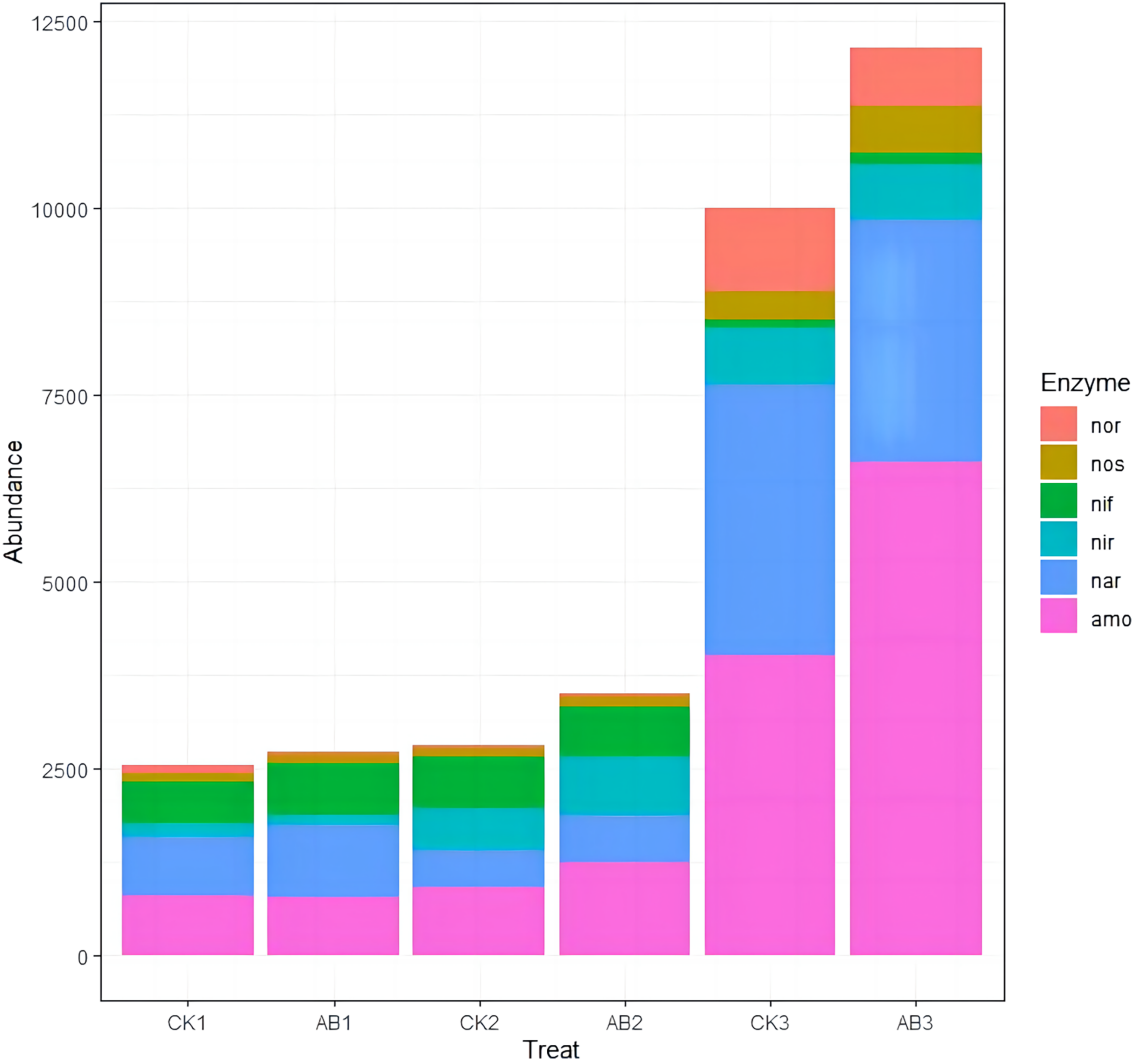
Changes of nitrogen transformation functional enzymes in swine manure compost. CK: The control group of swine manure composting; AB: Add 1% compound microbial inoculum in the swine manure composting. The numbers 1, 2 and 3 correspond to the mesophilic phase (day 1), thermophilic phase (day 2), and maturation phase (day 22) of swine manure composting, respectively.

The key processes involved in nitrogen transformation function enzymes in compost include ammonification, nitrification, denitrification and nitrogen fixation. In this study, metagenomic sequencing functional annotation analysis results showed that ammonia monooxygenase (amo) was increased, while *amoA* gene was decreased, which might be due to the inhibition of high temperature on amo enzyme activity (Figure 4). The denitrification process of compost was catalysed by nitrate reductase (nar), nitrite reductase (nir), NO reductase (nor) and N_2_O reductase (nos). With composting, nos and nir showed an increasing trend, while nar and nor decreased first and then increased. Nar was involved in the first step of denitrification (NO_3_^−^‐N → NO_2_^−^‐N), and its content decreased in the thermophilic phase, but increased significantly in the maturation phase. The relative abundance of nor in AB group was lower than that in CK group at day 1 (mesophilic phase), day 2 (thermophilic phase), and day 22 (maturation phase) of three different composting phases. The content of nitrogenase (nif) decreased significantly in the maturation phase. In general, AB group nif and amo were more abundant than CK group and adding compound microbial inoculum improved the abundance of amo and nif enzymes.

### Correlation analysis of nitrogen transformation genes, microbial community and environmental factors

**FIGURE 5:**
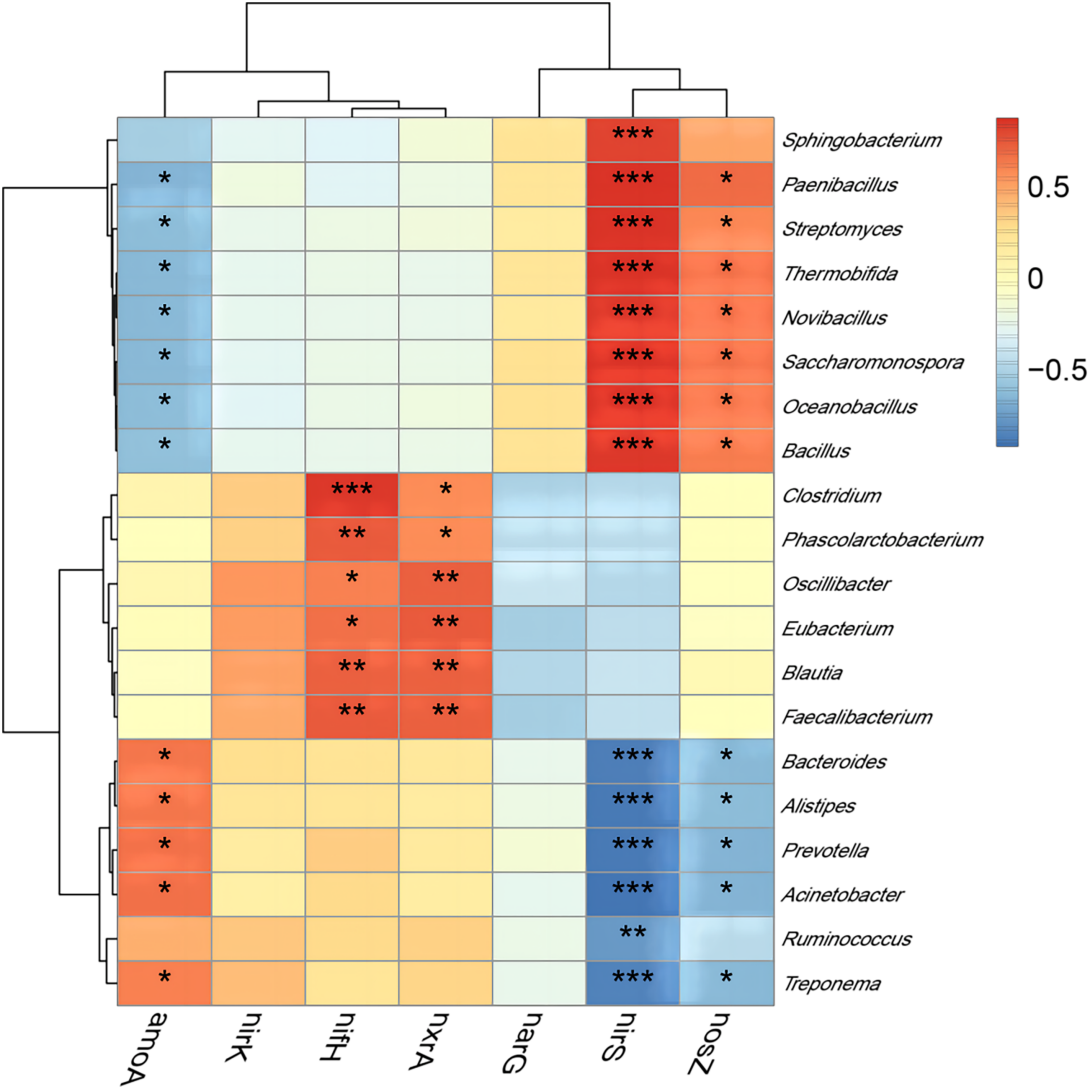
Correlation between nitrogen transformation genes and microbial community.

Correlations among nitrogen transforming genes and microbial community (genus level, top 20 abundance) are shown in Figure 5. The *nifH* and *nxrA* genes showed significant positive correlation with *Clostridium*, *Phascolctobacterium, oscillibacter*, *Eubacterium, Blautia* and *Faecalibacterium*, which were consistent with the results of microbial community changes and showed a trend of increase first and then decrease. These genus may be the main nitrogen‐preserving and nitrogen‐fixing microorganisms in compost. The *nirS* and *nosZ* genes involved in denitrification were significantly and positively correlated with *Paenochrobactrum*, *Streptomyces*, *Thermobifida*, *Novibacillus*, *Saccharomonospora*, *Oceanobacillus* and *Bacillus*, and their abundance were gradually increased during composting. The expression of *nirS* and *nosZ* genes were significantly negatively correlated with *Bacteroides*, *Alistipes*, *Prevotella*, *Acinetobacter* and *Treponema*, while *amoA* gene was significantly positively correlated with these bacteria, and the microflora of these species decreased gradually with the compost progress.

**FIGURE 6:**
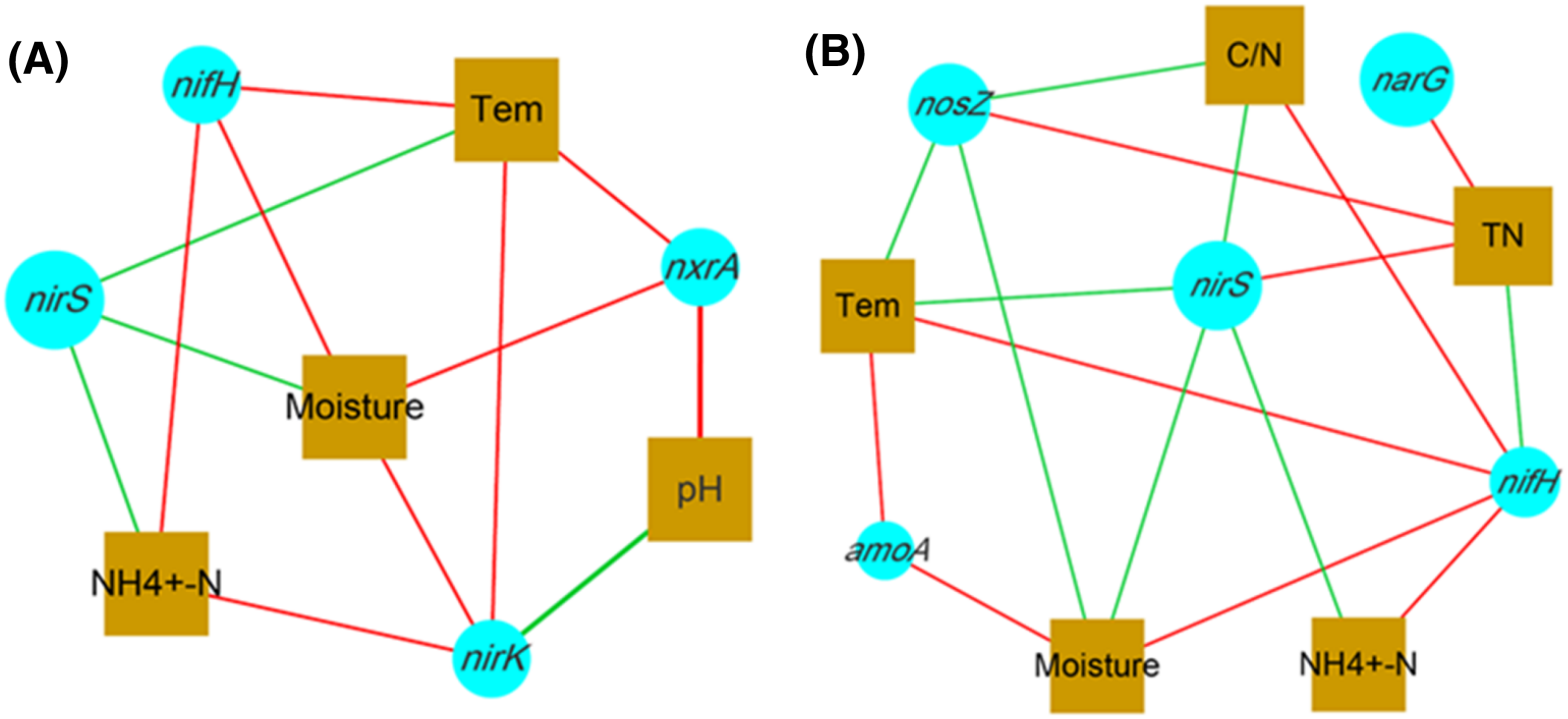
Correlation analysis of nitrogen transformation genes and environmental factors. (A) The control group of swine manure composting; (B) Add 1% compound microbial inoculum in the swine manure composting.

At the same time, the correlation between nitrogen transforming genes and environmental factors was further analysed by cytoscape. As shown in Figure 6, the red line and green line represented the positive correlation and negative correlation between nitrogen transforming genes and environmental factors, respectively. In the CK group (Figure 6A), *nxrA* exhibited a significant positive correlation with temperature, water, and pH levels. Similarly, relationships between *nifH*, *nirK* and temperature, water availability, ammonium nitrogen concentrations were significantly positive. In the AB group (Figure 6B), *amoA* displayed a significant positive correlation with temperature and moisture content. Additionally, *narG*, *nirS* and *nosZ* exhibited a significant positive correlation with total nitrogen levels. Furthermore, the nifH gene demonstrated a significant positive correlation with temperature, moisture content, C/N ratio as well as ammonium nitrogen concentrations.

**FIGURE 7:**
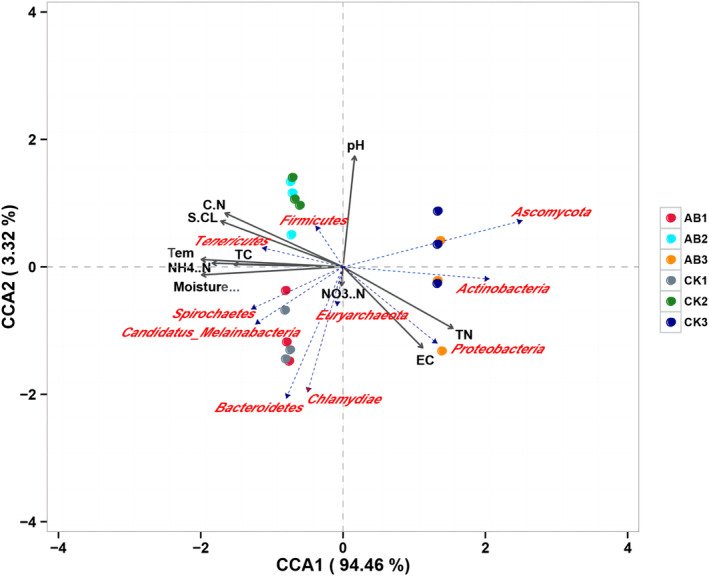
Phylum‐level analysis of environmental factors and microbial communities. CK: The control group of swine manure composting; AB: Add 1% compound microbial inoculum in the swine manure composting. The numbers 1, 2 and 3 correspond to the mesophilic phase (day 1), thermophilic phase (day 2) and maturation phase (day 22) of swine manure composting, respectively.

In this study, the influence of environmental factors on the top 10 microbial communities (phylum level) in abundance was assessed by CCA method (Figure 7). The results revealed that the selected environmental variables accounted for 97.78% of the species variation, with temperature, pH, NH_4_^+^‐N, TC, C/N ratio and cellulase activity exhibiting significant correlations with the microbial community. Moreover, alterations in TN content exerted a profound impact on *Proteobacteria* and *Actinobacteria* populations, while changes in pH and C/N ratio were closely associated with *Firmicutes*. Temperature, NH_4_^+^‐N, and water Caffected the activities of *Spirochaetae* and *Tenericutes*, while the NO_3_^−^‐N content was related to *Bacteroidetes* and *Euryarchaeota*. In the mesophilic phase of compost, moisture content and NH_4_^+^‐N played an important role in microbial activities. In the thermophilic phase, pH and C/N ratios played a pivotal role in shaping the microbial community dynamics. During the maturation phase, changes in microbial community were primarily influenced by TN levels and pH variations.

### Changes of pathogenic bacteria and ARGs in compost

**FIGURE 8:**
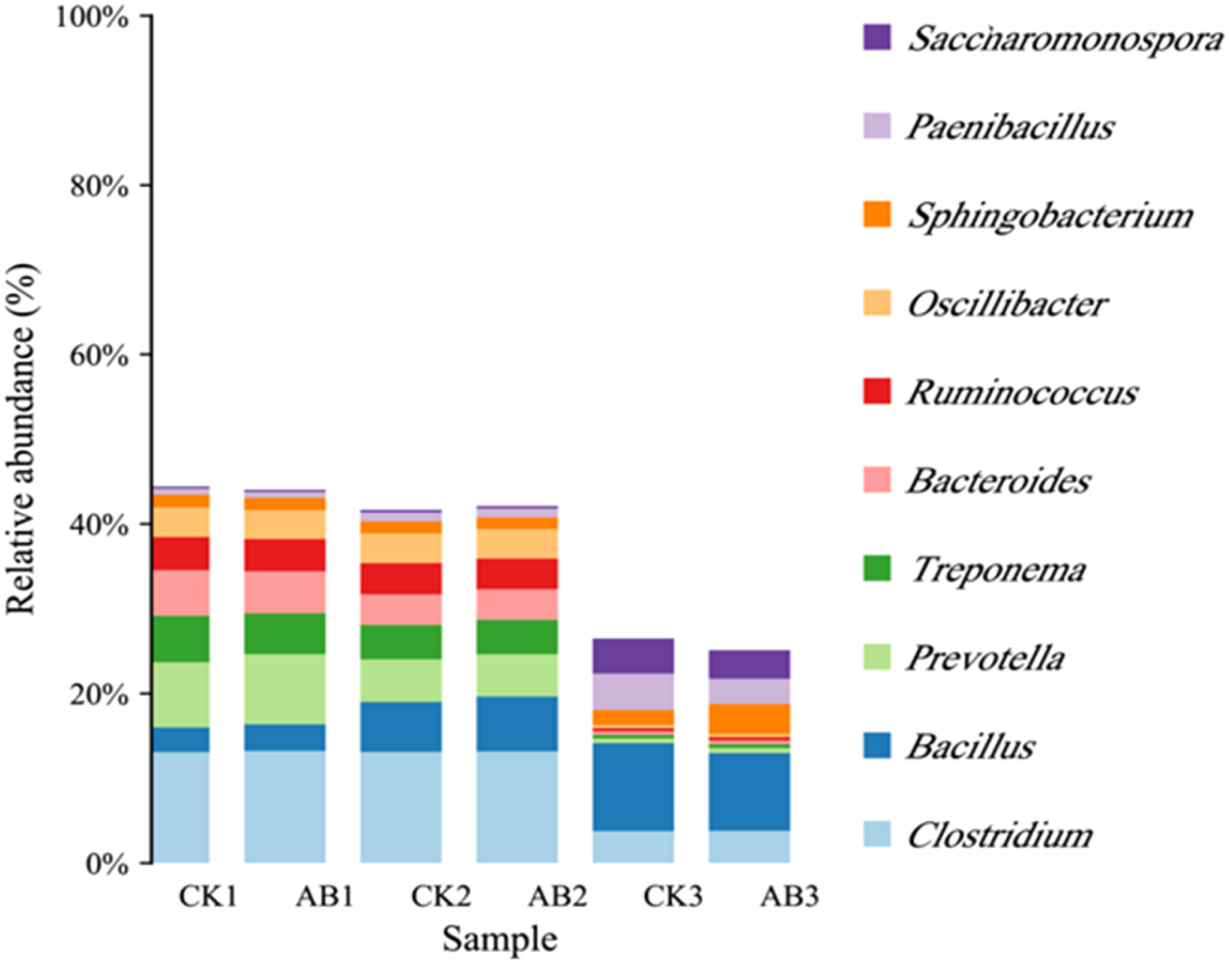
Changes of human pathogenic bacteria (HPB) during composting. CK: The control group of swine manure composting; AB: Add 1% compound microbial inoculum in the swine manure composting. The numbers 1, 2 and 3 correspond to the mesophilic phase (day 1), thermophilic phase (day 2) and maturation phase (day 22) of swine manure composting, respectively.

The present study employed metagenomic sequencing to further investigate the dynamics of pathogenic bacteria and antibiotic resistance genes (ARGs) during composting, aiming to explore the comprehensive enhancement effect of the compound microbial inoculum. According to the annotation results of PHI‐base database (Figure 8), the relative abundance of total pathogenic bacteria exhibited a decrease after composting. There has been a decrease in pathogenic bacteria in CK group of 40.35%, and a decrease in pathogenic bacteria in AB group of 42.92%, suggesting that compound microbial inoculum was helpful to the removal of pathogenic bacteria. Moreover, the reduction rates of pathogenic bacteria such as *Prevotella*, *Treponema*, *Ruminococcus* and *Bacteroides* in the CK and AB groups were all higher than 90%.

**FIGURE 9:**
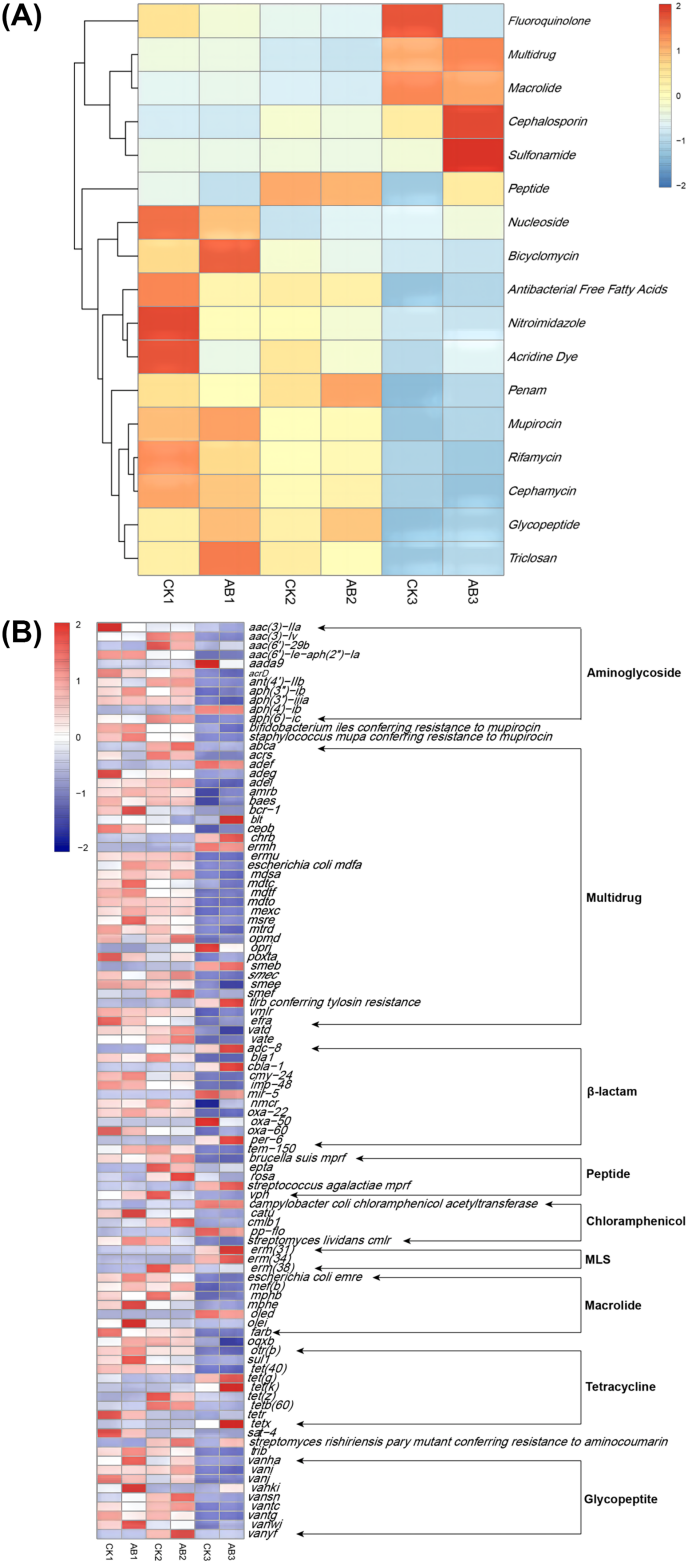
Changes in the relative abundance of antibiotic resistance genes (ARGs). (A) Changes in the relative abundance of ARGs carried by microorganisms; (B) Changes in relative abundance of ARGs subunits. CK: The control group of swine manure composting; AB: Add 1% compound microbial inoculum in the swine manure composting. The numbers 1, 2 and 3 correspond to the mesophilic phase (day 1), thermophilic phase (day 2) and maturation phase (day 22) of swine manure composting, respectively.

The sequencing data were compared with the CARD database to further analyse the changes in the relative abundance of ARGs and their subunits carried by microorganisms during composting. As shown in Figure 9, there was a significant decrease in the abundance of ARGs during the thermophilic phase of compost. In the maturation phase, the contents of *Nucleoside*, *Bicyclomycin*, *Mupirocin*, *Glycosides* and *Triclosan* were significantly reduced. The increase of some ARGs, such as *Sulfonamide*, *Cephalosporin*, *Multidrug*, *Macrolide* and *Fluoroquinolones*, may be caused by the dominant position of the microorganisms carrying these ARGs in the maturation phase of compost. The addition of the compound microbial inoculum was helpful to reduce some ARGs such as *Fluoroquinolone*, *Nucleoside* and *Nitroimidazole*. The *Fluoroquinolone* ARGs were reduced by 41.56% in the AB group, while those in the CK group were increased by 53.84 times (Figure 9A). Most of the ARGs subunits in the initial compost, such as *tetZ*, *tetR*, *tetL, rosB*, *ermB*, *sul1*, *mexX*, *adeG* and so forth, were reduced at the end of the compost. However, subunits such as *tetR* (*G*), *adeF, MIR‐5* and *oleD* proliferated (Figure 9B). At the end of composting, there was a significant difference in the proliferating ARGs between the compound microbial inoculum group and the control group, which might be due to the change of the potential host of ARGs caused by adding compound microbial inoculum. The above results indicated that the compound microbial inoculum could improve the efficiency of killing pathogenic bacteria and contribute to the removal of ARGs.

## DISCUSSION

In most cases, livestock manure is treated by composting. The measurement of temperature plays a crucial role in assessing the progress of composting, determining the level of microbial activity and monitoring organic decomposition (Huang et al., 2017). The composting device used in this study was similar to other studies (Duan et al., 2019). Due to the small volume of the device and rapid degradation of materials, the thermophilic phase was reached in the second day. NH_4_^+^‐N and NO_3_^−^‐N were important participants in the nitrification and denitrification processes and they were important for nitrogen transformation and nitrogen loss in compost (Azam, Müller, Weiske, Benckiser, & Ottow, 2002). The NH_4_^+^‐N concentration exhibited an initial sharp increase during the composting process, followed by a gradual decrease, which is consistent with findings reported in previous studies (Agyarko‐Mintah et al., 2017; Awasthi et al., 2018). The content of NO_3_^−^‐N first decreased and then gradually increased, because the initial high temperature would cause a large number of nitrifying bacteria to die, and then the nitrifying bacteria gradually recovered, and the content of NO_3_^−^‐N increased accordingly (Zhang, Luo, Li, Wang, & Li, 2018). The content of NH_4_^+^‐N, NO_3_^−^‐N and TN were significantly increased and the nitrogen loss was reduced by adding the compound microbial inoculum. Zhang et al. (2016) added ammonia‐oxidizing bacteria to chicken manure, which could convert more NH_4_^+^‐N into NO_3_^−^‐N, avoid excessive release of NH_3_ and reduce nitrogen loss. Qiu et al. (2021) added microbial inoculum made of bacteria capable of ammonizing, nitrifying, oxidizing nitrite, and fixing nitrogen to chicken manure compost, which converted more ammonia into total nitrogen and significantly reduce nitrogen loss. This study demonstrates that the incorporation of compound microbial inoculum exerts a positive influence on compost temperature elevation, accelerated water evaporation, as well as nitrogen preservation and fixation effects, which aligns with our previous investigation (Li, Cao, et al., 2020).

Metagenomic sequencing analysis showed that *Firmicutes* occupied the dominant position in both the mesophilic phase and thermophilic phase of swine manure composting, which was consistent with many research results (Guo et al., 2020; Lei et al., 2021; Wang et al., 2016). Adding microbial inoculum significantly changed the microbial community in swine manure compost. *Firmicutes* were reduced, but *Bacteroidetes* and *Actinobacteria* were increased during composting's maturation phase, as verified by genus‐level analysis.

*Paenibacillus* and *Pseudogracilibacillus* of *Firmicutes, Pseudomonas* and *Alkalilimnicola* of *Proteobacteria* were the most abundant bacteria in the control group at the maturation phase, while after the addition of microbial inoculum, the genus of *Bacteroidetes* (*Sphingobacterium*), *Actinobacteria* (*Brachybacterium*) and *fungi* (*Aspergillus*, *Acremonium*) were dominant. Studies have shown that *Proteobacteria* and *Actinobacteria* in the compost maturity stage will gradually replace *Firmicutes* as the dominant flora (Liu et al., 2015). In our previous study, *16S rRNA* sequencing also showed that adding compound microbial inoculum resulted in the decrease of *Firmicutes* and the increase of *Bacteroidetes* in the maturation phase of composting (Li, Cao, et al., 2020).

The *amoA* gene could initiate the first step of nitrification to oxidize ammonia to nitrite (Xu et al., 2018). During composting, its change would affect the contents of NH_4_^+^‐N and NO_3_^−^‐N. Adding the compound microbial inoculum significantly increased the abundance of *amoA* and *nxrA* genes, indicating that it could promote the nitrification process of composting to a certain extent in this study. The abundance of denitrifying genes (*narG*, *nirS*, *nirK* and *nosZ*) exceeded that of nitrifying genes (*amoA*, *nxrA*), suggesting a higher level of activity in the denitrification process compared to the nitrification process. Yu et al. (2020) found the same results in compost inoculated with cellulose‐degrading microorganisms. The addition of compound microbial inoculum was found to suppress the denitrification process and subsequently reduce nitrogen loss caused by N_2_, N_2_O and NO release, as evidenced by a lower abundance of *nirS* and *nosZ* at the end of composting in comparison to the control group. Researchers have found that exogenous microbial inoculum may affect the abundance of functional genes for nitrogen transformation by changing the microbial community and environmental factors (Guo et al., 2019; Huang et al., 2017). There is a negative correlation between some *Bacteroidetes* genes and the *nirS* and *nosZ* genes, and adding compound microbial inoculum improved the abundance of *Bacteroidetes* at the maturation phase of compost, which might be the reason for the low abundance of *nirS* and *nosZ* genes. The *nifH* gene is a specific marker gene of nitrogen‐fixing microorganisms (Meng, Zhou, Wu, Wang, & Gu, 2019), and the abundance changes of *nifH* gene can reflect the changes of nitrogen‐fixing microorganisms and the strength of nitrogen‐fixation effect during composting (Yin et al., 2018). Hu et al. (2021) found that microbial inoculation could stimulate the activities of nitrogen‐fixing bacteria community, thus increasing the abundance of *nifH* gene during composting. In this study, the abundance of *nifH* gene was kept high during the mesophilic phase and thermophilic phase of compost, especially in the compound microbial inoculation group, the abundance was about 4 times that of the control group, which indicated that the compound microbial inoculation could improve the nitrogen fixation of compost microorganisms. The analysis of functional enzymes further verified the above results, and adding compound microbial inoculum improved the abundance of amo and nif enzymes. In addition, the *nifH* and *nxrA* genes were significantly and positively correlated with four genus (*Clostridium*, *Phascolarctobacterium*, *Eubacterium* and *Faecalibacterium*) of Class Clostrida from *Firmicutes*, which may be the main nitrogen‐preserving and nitrogen‐fixing microorganisms in compost. The *nirS* and *nosZ* genes involved in denitrification were mainly positively correlated with three genus of *Actinobacteria* and two genus of *Firmicutes*, whose abundance gradually increased during composting.

The results of our study showed that the composting removed most of the pathogens, and the removal rate of the pathogens by adding the compound microbial inoculum reached 42.92%. (Cui, Wu, Zuo, & Chen, 2016) found that the removal rate of pathogens reached 30.4% when straw biochar was added into chicken manure compost. At the same time, we analysed ARGs and their subunits and found that most ARGs were also removed. Previous studies in our laboratory had shown that pathogenic bacteria may affect the abundance of ARGs, and the related ARGs carried by pathogens were also efficiently removed when the pathogens were removed (Li, Cao, et al., 2020). At the end of composting, there was a significant difference in the proliferating ARGs between the compound microbial inoculum group and the control group, which might be due to the change of the potential host of ARGs caused by adding compound microbial inoculum. The study of (Chen et al., 2022) proved this view.

The addition of microbial inoculum could increase temperature, accelerate water loss, and promote the accumulation of NH_4_^+^‐N, NO_3_^−^‐N and TN during swine and chicken manure composting. It also changed the microbial community abundance in the maturation phase of compost, resulting in the decrease of *Firmicutes* and the increase of *Actinobacteria* and *Bacteroidetes*. The compound microbial inoculum can enhance the nitrification and nitrogen fixation dominated by *amoA*, *nxrA* and *nifH*, and inhibit the denitrification of *nirS* and *nosZ* by influencing microbial community abundance and environmental variables such as temperature, water and nitrogen in compost.

## AUTHOR CONTRIBUTIONS

**Rui Cao:** Data curation (equal); formal analysis (equal); writing – original draft (equal). **Yihao Huang:** Writing – review and editing (equal). **Ruyu Li:** Writing – review and editing (supporting). **Ke Li:** Writing – review and editing (supporting). **Zhuqing Ren:** Conceptualization (lead). **Jian Wu:** Conceptualization (lead); writing – original draft (lead); writing – review and editing (lead).

## CONFLICT OF INTEREST STATEMENT

The authors declare no conflict of interest.

## Data Availability

The metagenomic sequencing raw data reported in this study are available in NCBI SRA with the accession number PRJNA684647: https://www.ncbi.nlm.nih.gov/bioproject/684647.
